# Predictors of applicant pool racial and ethnic diversity among physician assistant education programs: a national cross-sectional cohort study

**DOI:** 10.1186/s12909-023-04500-0

**Published:** 2023-07-18

**Authors:** Trenton J. Honda, Daytheon Sturges, Daphne C. Mills, Cynthia X. Yuen, Ryan W. Chitwood, José E. Rodríguez

**Affiliations:** 1 School of Clinical and Rehabilitation Sciences, Bouvé College of Health Sciences, Northeastern University, Boston, MA USA; 2 Department of Family Medicine, MEDEX Northwest, University of Washington School of Medicine, Seattle, WA USA; 3 Physician Assistant Education Association, Washington DC, USA; 4 National Education Association, Washington DC, USA; 5 Synovus, Columbus, GA USA; 6 Office of Health Equity, Diversity, and Inclusion, University of Utah Health, Salt Lake City, UT USA; 7 Department of Family and Preventive Medicine, University of Utah School of Medicine, Salt Lake City, UT USA

**Keywords:** Physician Assistant/Associate, Race/Ethnicity, Underrepresented minority, Medical Education, Matriculation.

## Abstract

**Background:**

Numerous studies have demonstrated that the increasing racial and ethnic diversity of the US population benefits from access to healthcare providers from similarly diverse backgrounds. Physician assistant (PA) education programs have striven to increase the diversity of the profession, which is predominantly non-Hispanic white, by focusing on admitting students from historically excluded populations. However, strategies such as holistic admissions are predicated on the existence of racially and ethnically diverse applicant pools. While studies have examined correlates of matriculation into a medical education program, this study looks earlier in the pipeline and investigates whether applicant – not matriculant – pool diversity varies among PA programs with different characteristics.

**Methods:**

Data were drawn from the 2017–2018 Central Application Service for PAs admissions cycle. Applications to programs with pre-professional tracks and applicants missing race/ethnicity data were excluded, resulting in data from 26,600 individuals who applied to 189 PA programs. We summarized the racial and ethnic diversity of each program’s applicant pools using: [1]the proportion of underrepresented minority (URM) students, [2]the proportion of students with backgrounds underrepresented in medicine (URiM), and [3]Simpson’s diversity index of a 7-category race/ethnicity combination. We used multiple regressions to model each diversity metric as a function of program characteristics including class size, accreditation status, type of institution, and other important features.

**Results:**

Regardless of the demographic diversity metric examined, we found that applicant diversity was higher among provisionally accredited programs and those receiving more applications. We also identified trends suggesting that programs in more metropolitan areas were able to attract more diverse applicants. Programs that did not require the GRE were also able to attract more diverse applicants when considering the URM and SDI metrics, though results for URiM were not statistically significant.

**Conclusions:**

Our findings provide insights into modifiable (e.g., GRE requirement) and non-modifiable (e.g., provisionally accredited) program characteristics that are associated with more demographically diverse applicant pools.

## Introduction

As the United States continues to become more racially and ethnically diverse, those from underrepresented minority (URM) and underrepresented in medicine (URiM) backgrounds make up a growing proportion of the population. As of 2019, 1.3% identify as American Indian or Alaskan Native, 5.9% identify as Asian, 13.4% identify as Black or African American, 0.2% identify as Native Hawaiian or Pacific Islander, 18% identify as Latino, Hispanic or of Spanish origin, and the remaining 60.1% identify as non-Hispanic white [1]. Because racial concordance between provider and patient is associated with better health outcomes, and higher satisfaction, there is great benefit to urgently increase the diversity of the physician assistant (PA) pool [2–5].

The current medical education workforce, however, does not reflect national diversity. Among 2019 PA grads: 0.5% are American Indian or Alaskan Native, 8.6% are Asian, 3.2% are Black or African American, 1.0% are Native Hawaiian or Pacific Islander, and 71.7% are white. These data, however, do not include the ethnicity categories that are recognized by the census (Hispanic or Latino/a/x, and non-Hispanic or Latino/a/x), and it is likely that the white group includes at least some Latino/a/x or Hispanic physician assistants (PAs) [6].

To increase the diversity of the health care workforce, we must first increase the diversity of the student body and, before that, the diversity of the applicant pools from which programs choose future practitioners. While many programs, such as the University of Utah PA program, have made significant strides by increasing multiple dimensions of diversity (e.g., age, racial and ethnic background, socioeconomic status) of the students in their program, the rate at which PA programs graduate students from historically excluded backgrounds remains unacceptably low [5], with a recent study finding that between 2014 and 2019, PA programs only 3.5% of PA program graduates were URM, and only 6.4% were Hispanic or Latino/a/x ethnicity [6, 7]. Among the strategies used, holistic admissions and universal faculty commitment to support diversity are major drivers for their success. Other research among PA students has shown that applicants who are URiM, older, men, and those with lower total or science GPAs or missing prerequisites are less likely to matriculate when compared to all other potential students, and this disparity is exacerbated when the Graduate Record Examinations (GRE) is a required application component [8, 9]. Recent research by Honda et al. (2021) has identified the importance of the number of programs to which a student applies on their likelihood of matriculation. One barrier to diversifying the student body that this study identified is that, on average, URiM students apply to fewer programs than their non URiM peers, with likely detrimental impacts on their likelihood of matriculation [10]. No matter how determined to implement diversity-boosting practices like holistic admissions, medical education programs are unable to admit diverse classes if they do not have first attract a diverse applicant pool. While this burgeoning literature has begun to identify the correlates explaining the differential likelihood of matriculation among applicants by race, to date, no studies have sought to identify program characteristics that are associated with diversity in the applicant pool. To better understand flows of diversity into the health professions, we examined the relationship between PA program characteristics and the diversity of their applicant pools.

## Methods

### Data sources and institutional review board

This is a secondary analysis of a data set including applicants to the 2017–2018 admissions cycle of the Central Application Service for Physician Assistants (CASPA), which is utilized by over 95% of accredited national PA programs. Students who did not list their race or ethnicity and programs with a pre-professional track were excluded, resulting in 26,600 applicants who applied to 189 PA programs. Access to the CASPA dataset was provided by the Physician Assistant Education Association (PAEA), and all participant identifying information was removed prior to PAEA providing the data for analysis. This research was determined to be exempt (non-human-subjects research) by the Institutional Review Board and all methods were carried out in accordance with relevant guidelines and regulations.

### Outcomes of interest

Outcomes of interest included percent of applicants to a program with (1) URM background, (2) URiM background, and (3) program-specific Simpson’s Diversity Index (SDI) [11]. Applicants were considered URM if they self-identified as Hispanic/Latino/a/x or any non-white race, whether alone or in combination with any other races. Applicants were considered URiM if they self-identified as Hispanic/Latino/a/x, or any non-Asian and non-white race, whether alone or in combination with any other races. For each program, we calculated the percentage of applicants from URM and URiM backgrounds as the dependent variable.

We also calculated for each program the SDI. The SDI originated in biology and economics but has been used in education research as well, and is defined as the probability that two individuals taken at random from the dataset of interest represent different racial or ethnic groups [12]. The index is described by the following equation:

SDI = 1 - (Σx^2^).

where *x* is the proportion of individuals in each race/ethnicity group. Each applicant was classified as one seven mutually exclusive groups: single-race Native American, Asian, Black, Native Hawaiian/Pacific Islander, white, Hispanic (of any race), or multiracial. SDI scores for each program were then calculated by summing the squared proportions for each group and subtracting from 1. Possible SDI scores range from 0 to 1. For example, within a given university, if the proportion of racial/ethnic groupings were as follows: Native American (5%), Asian (7.5%), Black (10%), Native Hawaiian/Pacific Islander (12.5%), white (15%), Hispanic/Latino/a/x (17.5%) or multiracial (32.5%), then we can calculate the probability of two randomly selected individuals representing the same racial/ethnic group as ((0.05)^2^ + (0.075)^2^ + (0.10)^2^ + (0.125)^2^ + (0.15)^2^ + (0.175)^2^ + (0.32.5)^2^ ) = 0.1925, while the probability of two randomly selected individuals representing two different racial or ethnic groups would be calculated as 1-0.1925, or 0.8075. The SDI is particularly useful, as it accounts for the impact that individual or several categories with disproportionately large proportions have on reducing diversity [12].

### Independent variables of interest

The primary predictors of interest included variables for which we *a priori* believed may be associated with increasing or decreasing diversity in the applicant pool. These included (1) Accreditation Status (Provisional, Continuing, or Probation), (2) Academic Health Center Status (binary), (3) Public versus private institution (binary), (4) GRE required for admission (binary), (5) the number of applicants, (6) the number of matriculants, and (7) the Urbanicity of the institution as determined by its Rural-Urban Continuum Code (RUCC; 1 = Metro area with population > 1 million; 2 = Metro area with population 250,000–1 million, 3 = Metro area with population < 250,000, and 4–9 = Non-metro area).

### Statistical analysis

We summarized data using descriptive statistics. We further employed individual multiple linear regression models for each of our outcomes of interest (% URM, % URiM, and SDI) to estimate associations between program characteristics and measures of program diversity. Effect estimates were presented as beta-coefficients and 95% confidence intervals, with the alpha value set *a priori* to 0.05. All statistical analyses were conducted with R version 3.6.1.

## Results

Table 1 shows the demographics characteristics of the programs in our study. Out of 189 programs that met inclusion criteria, the majority (66.7%) had an accreditation-continued status, just under a third (32.8%) were housed at academic medical centers, around half required the GRE (54.6%) and the majority were located in metro areas with populations > 1 million.

**Fig. 1:**
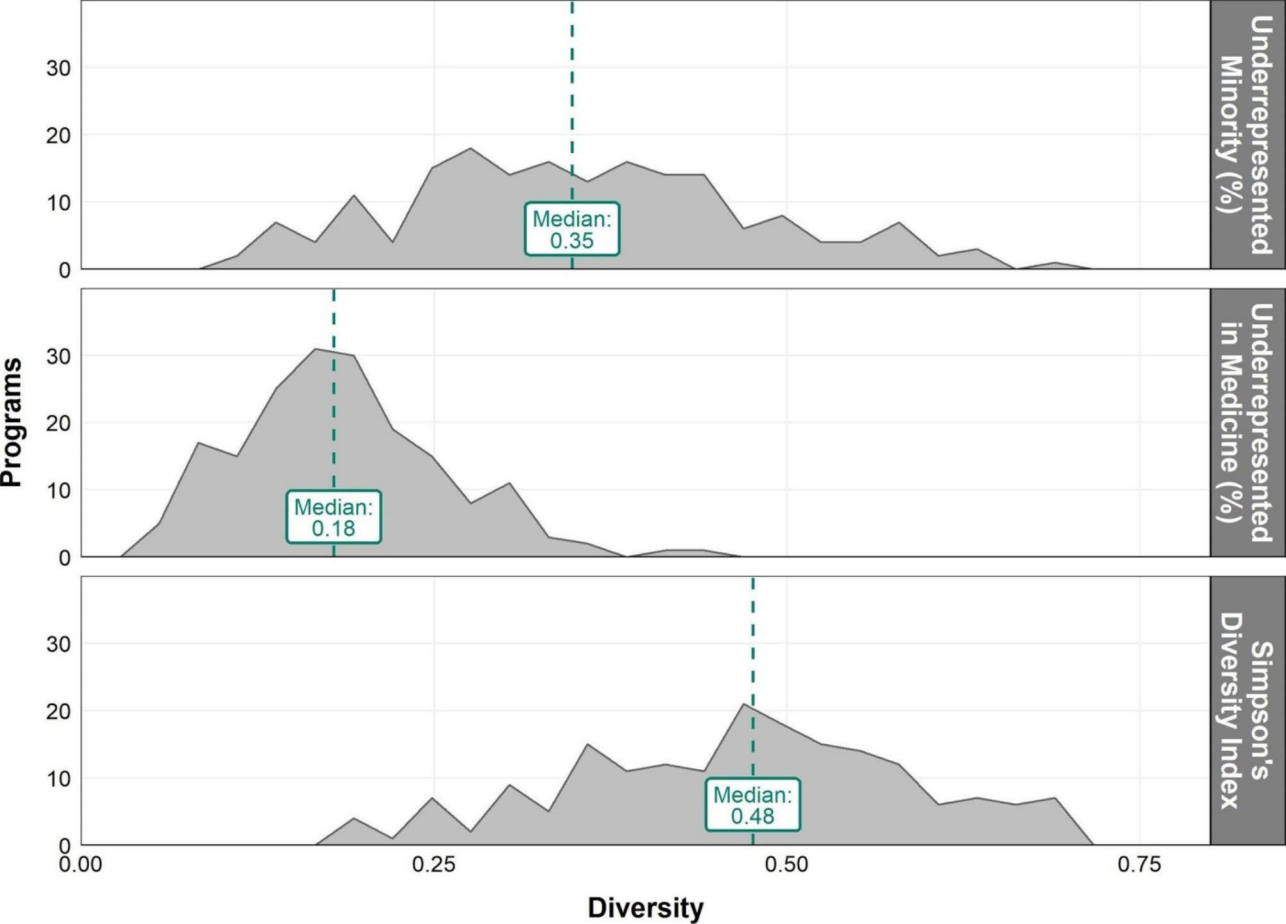
Distribution of racial/ethnic diversity outcomes in the study cohort

Figure 1 shows the distribution of diversity outcome metrics across the programs in our study. The proportion of URM and URiM students applying varied significantly across programs, with the median URM applicant proportion being 35%, compared to a much lower URiM of 18%. The median SDI for the study cohort was 0.48, which indicates that the median probability of two randomly selected individuals representing two different racial or ethnic groups within each school in our cohort was 48%.

**Table 1:** Program Characteristics

Program Characteristics	n (%)
**Accreditation Status**	
- Continuing	122 (66.7)
- Provisional	40 (21.9)
- Probation	20 (10.9)
**Academic Health Center Status**	
- Yes	60 (32.8)
- No	123 (67.2)
**Institution Type**	
- Public Institution	60 (32.8)
- Private Institution	121 (66.1)
**GRE Required**	
- Yes	100 (54.6)
- No	83 (45.4)
**Rural-Urban Continuum Code**	
- RUCC 1 (Metro area, pop. > 1 M)	100 (54.6)
- RUCC 2 (Metro area, pop. 250 K-1 M)	50 (27.3)
- RUCC 3 (Metro area, pop. < 250 K)	17 (9.3)
- RUCC 4–9 (Non-metro area)	16 (8.7)

**Fig. 2:**
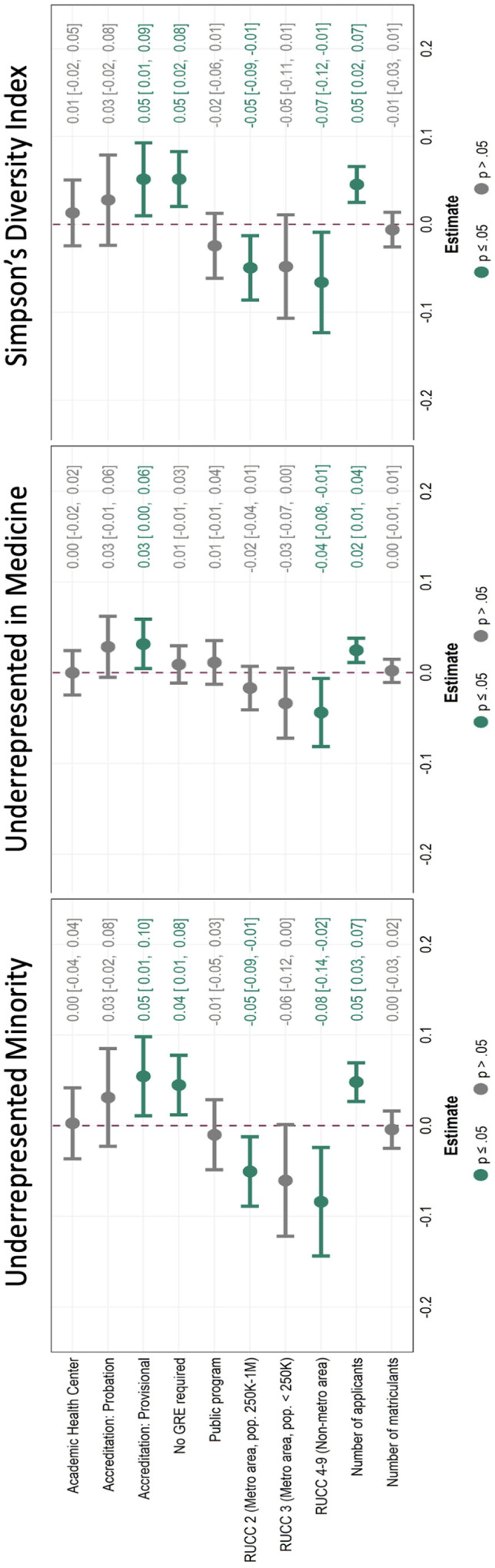
Associations between applicant pool diversity metrics and program characteristics

Figure 2 shows the results of our multiple linear regression analyses associating program characteristics with applicant pool metrics of diversity. Across all three metrics of diversity accreditation provisional status (as compared to accreditation-continued) and higher applicant number were positively and significantly associated with increased applicant pool diversity. Likewise, programs in non-metro areas were associated with decreased diversity across all three outcomes of interest. Lack of a GRE requirement was significantly associated with increased URM% and SDI, but not URiM, while programs in metropolitan areas with populations of between 250,000 and 1 million were inversely associated with URM% and SDI, but not URiM. Number of matriculants, public versus private status of the sponsoring institution, and whether the sponsoring institution was an academic health center was not significantly associated with any outcomes of interest.

## Discussion

Our study is the first to explore associations between PA program characteristics and applicant pool racial/ethnic diversity in a large national cohort of United States programs. We found that programs with a larger number of applicants, without a GRE requirement, and those with provisional accreditation status were associated with higher proportions of URM and URiM applicants, and with a larger SDI, indicating greater applicant diversity. Programs in smaller metropolitan areas (and particularly the smallest metropolitan areas) were associated with significantly smaller proportions of URM and URiM applicants, and smaller SDIs.

Various insights and inferences can be drawn from the literature concordant with the study’s findings. The implications associated with these results are considerable as intentional medical education programs seek to diversify the applicant pool to achieve population parity with ever-evolving United States racial and ethnicity demographics. Achieving a more diverse health care workforce supports cultural humility as well as inclusive patient care for historically excluded populations. As the protectors of the public trust, health education programs have an opportunity to decrease health disparities, achieve reductions in health spending, and defray health systems waste [13, 14].

Provisional programs attracting a more racially and ethnically diverse applicant pool may be explained by considering the program’s racial/ethnicity composition. In the case of a newly accredited PA program, URM/URiM applicants may view their chances for acceptance as more favorable as there are zero or few matriculated classes upon which to draw a conclusion regarding a program’s diversity priority. Past studies support that applicants from URM/URiM backgrounds assess a program’s current diversity [8, 15]. For example, PA programs with little to no URM/URiM students and/or faculty representation experience recruitment and retention issues regarding historically excluded student populations [15]. Considering the importance of established diversity in choosing which PA programs to apply, it could be the lack of historical racial/ethnicity compositional data that attract URM/URiM applicants to provisionally accredited programs. Yuen posits “that the diversity – and perhaps especially the racial and ethnic diversity – of a PA program’s students and faculty may be a more salient consideration for minority students.” [15] Provisionally accredited PA programs have yet to establish themselves as one that is viewed through either an inclusionary or exclusionary lens regarding belonging from the URM/URiM applicant perspective.

Programs that do not require the GRE are demonstrating inclusive excellence by removing systemic barriers and promoting equitable practices. Most PA programs require or recommend minimum GRE scores to qualify or disqualify applicants. This practice does not align with the test developer’s (Educational Testing Services) formal guidance for use of scores [16]. The GRE requirement and misuse of scores are both hindrances to developing a more diverse PA workforce [8, 16]. Beyond PA school admissions, standardized tests also serve as barriers for applicants to both medical and physical therapy programs [17]. For PA programs recommending the GRE, matriculation decreased among URM/URiM applicants who did not submit scores [8]. Therefore, it can be deduced that URM/URiM applicants will most likely choose to apply to programs in which they have higher odds for successful matriculation without the barrier of a standardized test requirement.

Larger applicant pools seem to attract more diversity. The medical school literature supports that “a large diverse applicant pool is needed to ensure the appropriate candidates can be chosen. . .” [18] In order to bolster diversity, schools should benchmark data from comparable regional and/or national academic institutions. This practice leads to determining the estimated size of the applicant pool needed to achieve more diverse student applicants and matriculants [18]. Once implemented, ongoing sustainability should be prioritized by deploying strategic marketing that highlights student/faculty diversity and leadership in order to inculcate a sense of belonging [18, 19].

Metropolitan and urban areas tend to have more diverse populations, though rural areas are currently experiencing growing racial/ethnicity diversity. However, historically, rural areas have been perceived to be less diverse and more homogenous regarding their respective populations. Previous studies support that when making college decisions, Black American and Hispanic American students prioritize both campus and neighboring community racial diversity [15]. Similarly, diversity data regarding local populations have a positive concordance with URM/URiM student numbers in both dental and physical therapy programs [17]. Coplan et al. demonstrated a positive correlation between the matriculation of Hispanic/Latino/a/x PA students in the Western United States, while noting a negative correlation with Midwest programs consistent with local population demographics [20]. Therefore, perceived lack of diversity regarding local communities (e.g., rural areas) may serve as a barrier to attracting URM/URiM PA program applicants [17]. Additionally, it is possible that racial/ethnic diversity in the applicant pool is impacted by the geographic diversity of the applicant pool catchment area for each program. Future research could investigate the diversity of the unique catchment area for each program (some of which are national/international, while others are more local), the degree to which this is similar or divergent from the community in which the PA program is physically situated, and whether this is these factors impact the applicant pool.

PA school applicants from historically excluded populations have related financial barriers in their pursuits for higher educational attainment. Thus, prior research has shown more URM/URiM applicants eventually enrolling in public-sponsored PA programs when compared to private-sponsored PA program enrollment [17]. Mission statements may also attract diverse applicants, especially those statements that are also manifest in a PA program’s holistic admissions process [21]. Of note, diversity has been shown to be regularly prioritized in public PA program mission statements [22].

Understanding factors that influence which programs prospective URM/URiM health care providers decide to apply to is paramount to increasing the diversity of medical providers. Though some contributors are not easily modified, PA programs can immediately begin to dismantle existing systemic barriers and re-visit their mission statements. PA programs can also begin to develop strategies that highlight their commitment to recruiting and retaining URM/URiM students while also inculcating an inclusive atmosphere of belonging and support.

### Strengths/Limitations

Our study has several important limitations that must be noted. First, while we analyzed national CASPA datasets which capture the vast majority of PA school applicants in the US, approximately 5% of US PA programs do not utilize the CASPA application system and as such our findings may not be universally generalizable. Second, the CASPA data is largely dependent upon applicant self-report of data. As such, it is possible that data on covariates may be reported in error or subject to reporting bias, resulting in residual confounding. Third, our study is cross-sectional and observational, factors which preclude causal interpretations. Fourth, we did not have information on other potential predictors of interest that likely impact the diversity of the applicant pool, such as program tuition [5], healthcare experience requirements, or the Historical Black Colleges and Universities (HBCU) status of the sponsoring institution. These weaknesses are counterbalanced by our use of robust national datasets which provide access to high quality predictor, outcome, and confounder information. Additionally, our datasets predate the COVID-19 pre-pandemic and the murder of George Floyd, and thus do not capture the impacts of these unprecedented global and national events on PA applicant data. More research is necessary to determine whether and to what extent applicant behavior has been affected by these events.

## Conclusions

Increasing racial and ethnic diversity in the health workforce is an ongoing challenge. As the gatekeepers to the profession, these training programs have rightly focused on admitting students from historically underrepresented backgrounds. However, most recent endeavors and research have centered around the question of who gets in and less is known about who applies, to which programs, and why. We found that programs with provisional accreditation, that receive more applicants, and that don’t require the GRE, are associated with higher diversity among PA applicants, but only public schools maintain the increased diversity during matriculation. Additional research should explore why each of these associations exist to identify mechanisms that programs can implement to increase the diversity of their applicant pools.

## Data Availability

The data that support the findings of this study are available from the Physician Assistant Education Association (PAEA) but restrictions apply to the availability of these data, which were used under license for the current study, and so are not publicly available. Data are however available from the corresponding author, Trenton Honda, upon reasonable request and with permission of PAEA.
